# Great ape communication as contextual social inference: a computational modelling perspective

**DOI:** 10.1098/rstb.2021.0096

**Published:** 2022-09-12

**Authors:** Manuel Bohn, Katja Liebal, Linda Oña, Michael Henry Tessler

**Affiliations:** ^1^ Department of Comparative Cultural Psychology, Max Planck Institute for Evolutionary Anthropology, 04103 Leipzig, Germany; ^2^ Institute of Biology, Leipzig University, 04103 Leipzig, Germany; ^3^ Naturalistic Social Cognition Group, Max Planck Institute for Human Development, 14195 Berlin, Germany; ^4^ Department of Brain and Cognitive Sciences, Massachusetts Institute of Technology, Cambridge, MA 02139-4307, USA

**Keywords:** communication, primates, social cognition, evolution, computational modelling

## Abstract

Human communication has been described as a contextual social inference process. Research into great ape communication has been inspired by this view to look for the evolutionary roots of the social, cognitive and interactional processes involved in human communication. This approach has been highly productive, yet it is partly compromised by the widespread focus on how great apes use and understand individual signals. This paper introduces a computational model that formalizes great ape communication as a multi-faceted social inference process that integrates (a) information contained in the signals that make up an utterance, (b) the relationship between communicative partners and (c) the social context. This model makes accurate qualitative and quantitative predictions about real-world communicative interactions between semi-wild-living chimpanzees. When enriched with a pragmatic reasoning process, the model explains repeatedly reported differences between humans and great apes in the interpretation of ambiguous signals (e.g. pointing or iconic gestures). This approach has direct implications for observational and experimental studies of great ape communication and provides a new tool for theorizing about the evolution of uniquely human communication.

This article is part of the theme issue ‘Revisiting the human ‘interaction engine’: comparative approaches to social action coordination’.

## 1. Introduction

When discussing the origins of human communication, Levinson and colleagues [1,2] introduced the idea of a human *interaction engine*. This metaphorical engine is assembled from a range of social-interactional parts that, when put together, enable uniquely human forms of communication, including conventional language. Each part was assumed to have deep roots in our evolutionary history and might therefore—in one form or the other—also be found in other primates. Inspired by these ideas, this paper introduces a computational model that specifies the role that social-interactional processes play in great ape and human communication.

What are the parts that the human interaction engine is built from? First and foremost, human communication is seen as intentional. Senders produce signals to convey intentions and receivers use these signals to infer the sender’s intentions [3–6]. As such, communication is deeply linked to reasoning about mental states. Signals, including conventional language, are used to express intentions but the link between signals and intentions is not rigid. There is always residual ambiguity that requires communicators to make additional (pragmatic) inferences—a second key feature of human communication. Such inferences are licensed by a set of assumptions that humans hold about the nature of communication and social interaction more broadly. One such assumption is that communication occurs within some form of common ground—a shared body of knowledge and beliefs that builds up during social interaction and serves as the background against which signals are interpreted [7,8]. Another assumption is that communication is cooperative such that senders choose their signals so that the receiver is more likely to infer the underlying intention [9]. The receiver takes this into account when interpreting the signal.

The engine assembled from these—and many other—parts is independent of any particular modality. Multi-modality is seen as the norm, not an exception in human communication. The system is also highly flexible. Sometimes a tiny hand gesture might be enough to get a message across; at other times, the same meaning might require a long, elaborate utterance comprised of multiple signals that are combined according to conventional rules (grammar). Or as Levinson & Holler [2] put it, ‘The system remains highly flexible, allowing us to shift the burden from words to gestures as required by the current communicative needs.’ Many roads lead to Rome in human communication and *what* works *when* depends on the social-interactional embedding. The system is also independent of the availability of conventional (or evolved) signals. Conventional language is assumed to rely on the engine in just the same way as non-conventional communication. New signals can be invented and understood on the spot and later even conventionalized into new languages [10–18].

The picture that emerges here provides an interesting starting point for an evolutionary research program because it decouples human communication from conventional language. The idea is that there is probably no direct link between the kinds of signals our ancestors used (which might be comparable to what we see in great apes) and human language. The link lies in *how* signals are used, that is, the social and cognitive underpinnings of communication. Once the interaction engine was in place, our ancestors started using and creating signals that, via intermediate proto-languages, evolved to become what we today see as conventional languages [19–23]. Thus, in addition to looking for structural features in animal communication that directly resemble aspects of conventional language (e.g. arbitrary sound-to-meaning mappings or combinatorial syntax [24–28]), comparative researchers can also ask which social-interactional processes underlie communication in other animals. In the next section, we will briefly summarize research in this tradition, with a focus on great ape communication.

## 2. A comparative approach to human language: the intentional nature of great ape communication

It is beyond the scope of this paper to give a comprehensive summary of existing research on primate communication. We will focus on two aspects that have received considerable attention in comparative research: signallers’ intentional signal production and receivers’ extraction of the intended meaning of a signal. We will show that research on these two aspects of great ape communication varies drastically depending on whether the focus is on vocal, gestural, or facial signals. To make matters worse, there are also marked differences between research on the production versus the perception or comprehension of signals.

To identify acts of intentional communication in great apes and other non-human primates, Leavens *et al*. [29] suggested a set of criteria derived from research on pre-linguistic communication in human infants [30]. These include the sender’s sensitivity to the presence of other individuals, visual orienting behaviour and monitoring of the receiver, the adjustment of signal use to the receiver’s attentional state and the use of attention-getting behaviours if receivers are not visually attending. Finally, senders are expected to continue signaling and to elaborate signal use in case initial communicative attempts fail.

There is now ample evidence that great apes are intentional communicators in that sense, not only in the gestural modality [31,32]. For example, several species of great apes adjust their signal use to the attentional state of the receiver and only deploy visual gestures if the receiver is attending [29,33]. They also wait for a response and persist in their communicative attempts and might even elaborate their gesture use if the receiver does not react [29,34,35]. Sumatran orangutans use gestures and also some facial expressions flexibly to achieve a variety of social goals [36,37]. Furthermore, wild chimpanzees are more likely to produce alarm calls when other individuals are unaware of a potential threat [38,39].

However, which and how many of the criteria for intentional communication are applied does not only vary across studies but also across modalities [31]. While intentional use is an integral part of defining a gesture, until more recently, this aspect was not considered important in vocal and facial research [40], resulting in the common but unjustified dichotomy between intentional gestures and emotional vocalizations and facial expressions [6].

The different theoretical and methodological approaches in vocal, gestural and facial research have serious downstream consequences for research on primate communication more broadly. Gesture researchers focus on the behaviour of the sender because of the importance of intentional signal production, while vocal and to a lesser extent also facial researchers focus on signal perception and how receivers extract a signal’s meaning. Vocal researchers, for example, frequently use playback experiments to study receivers’ reactions to a very specific call to identify the meaning or function of this call [41]. As a consequence, vocal researchers are interested in context-specific signals, with very specific meanings, while gesture researchers investigate the flexible use of one signal across different contexts and argue that the information conveyed by a gesture might differ depending on the context in which it is used. Gesture researchers further largely ignore context-specific signals because this would not fulfil the criterion of flexible usage, which is often considered an additional marker of intentional use [31,36].

Meaning is also conceptualized very differently across modalities, depending on whether the focus is on the signaler’s or receiver’s behaviour [40]. While gesture researchers focus on the message the signaler intends to communicate, vocal (and partly also facial) researchers focus on the ‘meaning’ extracted by the receiver [42,43]. As a consequence, it is difficult—if not impossible—to compare findings across modalities with regard to how non-human primates’ communicative interactions are shaped by contextual information and how they ‘make sense’ of others’ communicative attempts. Only more recently has there been some cross-fertilization in both vocal and gesture research. Vocal researchers report that some vocalizations are less context-specific than previously thought [44], while gesture researchers started to assign specific meanings to individual gestures [45,46].

Despite these recent developments, it is important to highlight that research on primate communication has almost exclusively used a uni-modal approach: the majority of research focused either on gestural, vocal or facial signals, and only very few studies investigated more than one signal modality simultaneously [47–51]. There are a number of different reasons why researchers artificially break up the communicative process into components and study each of them in isolation [52]. For example, researchers are trained in the theoretical approach and methods of their focal modality; methods used to study one modality (e.g. playback experiments) are not easily applicable to another modality.

There is, however, a deeper and more fundamental problem: we lack a theoretical account of how the different components integrate with one another. For human communication, Enfield [53], for example, proposed that composite utterances, incorporating multiple signals of multiple types, ‘[…] are interpreted through the recognition and bringing together of these multiple signs under a pragmatic unity heuristic or co-relevance principle, i.e. interpreter’s steadfast presumption of pragmatic unity despite semiotic complexity’. In other words, the recognition of each component’s (encoded) meaning is enriched by (the interpretation of) additional information, such as the meaning provided by the context in which this utterance is embedded. For primate communication, an equivalent theoretical account is still missing and many of the following questions remain unsolved. How do different signals relate to one another? That is, how does the combination of a gesture with another signal (e.g. gesture, facial expression or vocalization) change the meaning or usage of the initial gesture? What role does the social context play? Our goal for the rest of the paper is to sketch out such a theoretical account in the form of a computational model. As a first step, we will briefly introduce the Rational Speech Act (RSA) framework that formalizes some of the reasoning processes implied by the interaction engine and from which we took inspiration.

## 3. Computational models of inferential communication in humans

A core challenge for a multi-layered, multi-modal system is to specify how the different information sources—the aspects of the utterance and the context that relate to the message being communicated—flow together [53–56]. The RSA framework sees communication as a socially guided inference process [57,58]. A hypothetical receiver in the model is assumed to reason about the intention that underlies the sender’s production of an utterance in context.^1^ Importantly, the receiver assumes that the sender is communicating in a cooperative way, choosing utterances that are maximally informative for the receiver given the context. This assumption allows the receiver to go beyond the literal meaning of the words that are used and to make pragmatic inferences.

The RSA framework has been successfully used to model a range of language understanding phenomena as pragmatic inferences including scalar and *ad hoc* implicatures, non-literal language, politeness and vagueness, among others [57,59–63]. More recently, it has been used to predict how adults and children integrate different information sources to make inferences about what a sender is referring to [64]. In one study, Bohn *et al*. [65] measured children’s developing sensitivity to different information sources, for example, their linguistic knowledge or their sensitivity to common ground. Then they used an RSA-type model to predict what should happen when children are confronted with multiple information sources at once. When they compared these predictions to new experimental data, they saw a very close alignment between the two, both qualitatively and quantitatively. To learn more about the integration process itself, they formalized a range of alternative models that varied in their assumptions about which information sources children used and how they integrate them. They found that children’s behaviour was best predicted by a model that assumed rational integration of all available information sources. Interestingly, the integration process was best described as stable across development. That is, even though children might change in how sensitive they are to different information sources, the way they integrate them seems not to change as they develop. These studies illustrate how computational models can be used as a tool to study multi-layered communication.

For the model we describe below, we take inspiration from the RSA framework. The connection is mainly conceptual: we see communication as a socially guided inference process that relies on multiple, context-dependent information sources. There is, however, little structural overlap in terms of the implied cognitive mechanisms. In §6, we explore how the social reasoning processes that are structural characteristics of RSA can be used to explain differences between great ape and human communication when it comes to interpreting novel and ambiguous signals.

## 4. Formal models of primate communication

Our main goal in this paper is to formulate a computational model of great ape communication. We focus on the in-the-moment comprehension of communicative acts. We ask how a receiver makes inferences about the intentions of a sender based on information contained in the signals that make up an utterance, the relationship between communicative partners, and the social context. The process of in-the-moment comprehension has received little attention in previous modelling work in primate communication. We briefly review some of the earlier literature before laying out our approach.

Most formal work in primate communication has focused on modelling the production of different primate calls [66,67]. Though relevant for answering questions about the evolution of speech, this work does not help us understand the social-interactional nature of primate or ape communication. In a very ambitious project, Stuart Altmann^2^ [68] used stochastic models to predict the socio-communicative behaviour of rhesus monkeys (*Macaca mulatta*). He observed large groups of monkeys living on Cayo Santiago for two years with the goal to develop an ethogram of the species’ social behaviour. Next, he used his observations to define transitional probabilities between different behaviours. That is, he asked how well one can predict an individual’s behaviour if the previous behaviour (by the same or another individual) is known. He did this for pairs of behaviours, but also for longer sequences. Perhaps unsurprisingly, he found that the behavioural stream is not a random sequence of events, but that behaviours cluster in a systematic way. In a very broad sense, we take this as an inspiration to look for a wider set of determinants when trying to predict in-the-moment comprehension and reactions.

Arbib and colleagues [69–72] focused specifically on gestural communication. Their main goal, however, was to model the ontogeny of gestures. Their model shows how behavioural patterns can evolve into communicative gestures during direct, physical interaction. Given their specific aim, the authors saw the gesture as the sole cause of changes in the receiver’s behaviour. Comprehension is treated as an associative learning process during which the observation of a particular action becomes paired with a particular reaction (i.e. change in the receiver’s goal state). The result is a linear mapping between observing a gesture and producing an outcome. In our model, we loosen this assumption and take into account that multiple information sources influence the response to a gesture.

## 5. A computational model of chimpanzee communication

In this section, we introduce a Bayesian computational model of great ape communication. In contrast to standard statistical procedures (e.g. linear regression) that describe a particular dataset, our model describes the inference processes we assume to underlie great apes’ interpretation of communicative signals in context. These inference processes are built into the model structure and the model provides an account of the process that generated the data. Such a generative model can be used to predict and explain datasets (see below), but its main purpose is to provide a theoretical account of the phenomenon in question. In what follows, we first present a very general formulation of our model and then further specify it to capture a particular type of communicative interaction. We then evaluate the model based on an existing dataset.

**Figure 1.**
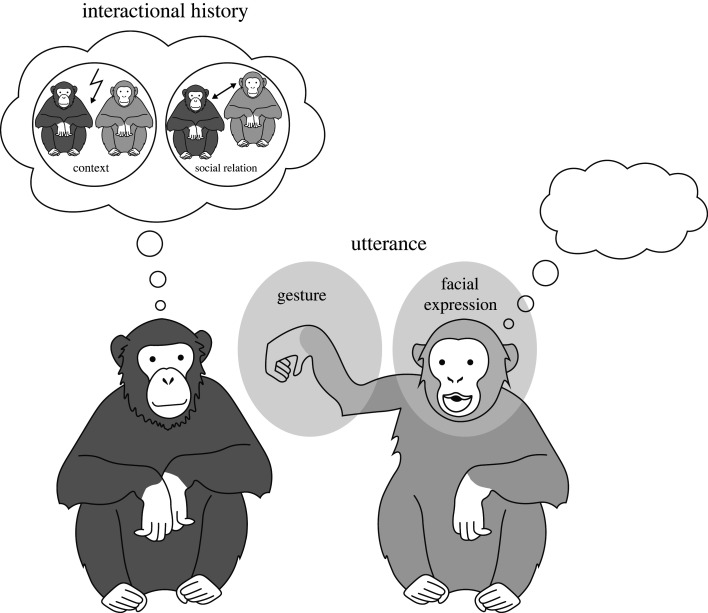
Schematic overview of the computational model. The sender (right) is producing an utterance and the receiver (left) tries to infer the intention of the sender based on the information sources available. The model takes in information provided by the utterance (gesture and facial expression) and the interactional history (immediate social context and dominance relation).

We see great ape communication as a contextualized social inference problem. That is, the sender produces an utterance that the receiver uses to make inferences about the sender’s intention (figure 1). Utterances can be composed of different types of signals coming from different modalities (e.g. gestures, vocalizations, facial expressions etc.). Inferences are contextualized in that not just the utterance, but also the social context of the utterance as well as the relationship between the sender and receiver influence the receiver’s interpretation. Thus, multiple information sources have to be integrated. We explore the hypothesis that this integration process occurs via a rational Bayesian procedure. This contrasts with the use of the term rational as describing a rule-based (i.e. logical) form of drawing conclusions. Here, we assume that the receiver’s a posteriori belief is optimal given the receiver’s prior beliefs and the constituent information sources they receive [73–75]. Given the simplicity of our model, we do not assume any limitations with respect to the cognitive resources that our communicative agents have at their disposal. However, our approach could easily be extended in this direction, for example, with resource-rational considerations [76]. The model is formally defined as

5.1 P(i∣u)∝P(u∣i)P(i),

with *P*(*i*|*u*) being the probability that the sender has intention *i* given utterance *u*. This decomposes into the likelihood of producing an utterance given an intention *P*(*u*|*i*) (e.g. raising one’s arm when wanting to be groomed) and the prior probability of having an intention in the first place *P*(*i*) (e.g. wanting to be groomed). This very general formulation can be used as a framework to evaluate different hypotheses about which social information sources contribute to the likelihood and the prior; that is, which information sources play an important role in great ape communication.

Next, we spell out one variant of the model, which was in part determined by the dataset that we had available for evaluation. As mentioned above, the general framework could be used with more, fewer, or different information sources. For the purpose of the current paper, the likelihood is defined by the semantics associated with a gesture, L(g,i), and a facial expression, L(f,i), which independently contribute to make up the utterance

5.2 $$P(u|i)=P(g,f|i)=L(g,i|\theta_{g})L(f,i|\theta_{\;f}).$$

Signals have ‘soft semantics’, that is, in contrast to a truth-functional (Boolean) semantics, we assume a probabilistic mapping between a signal and an intention (defined by the parameters *θ*_*g*_ and *θ*_*f*_ [77]; where *θ*_*g*_ is the strength of association between the gesture and the intention and *θ*_*f*_ that of the facial expression and the intention). The utterance is contextualized by the prior probability of the intention, *P*(*i*), which we take to be a function of the context, and the social relation between individuals, *P*(*i*|*c*, *s*)

5.3 $$P(i)=P(i|c,s)=\rho_{c}\rho_{s}.$$

The direction and strength of the context and social relation components are defined by the parameters *ρ*_*c*_ and *ρ*_*s*_ (where *ρ*_*c*_ denotes the association between the context and the intention and *ρ*_*s*_ that between the social relation and the intention). In the example below, we provide more information about the interpretation of these parameters.

To evaluate the model, we used it to predict the outcome of communicative interactions between semi-wild-living chimpanzees (*Pan troglodytes*). The data are taken from the study by Oña *et al*. [50] in which the authors observed two groups of chimpanzees (72 individuals) living in the Chimfunshi Wildlife Orphanage Trust in Zambia. They investigated if signal combinations were used in different contexts and/or elicited different responses compared to signals used alone. For every communicative interaction, they recorded the signals the sender produced, the context in which they were used and the reaction of the receiver. More specifically, they coded the type of manual gesture using a form-based coding scheme, differentiating between morphological configurations of the joints of the arm, hand and fingers. Using this procedure, they identified two frequently occurring gesture types: *stretched-arm*, consisting of an extended arm with both the arm and hand stretched, and *bent-arm*, with either hand or forearm bent and the back of the hand or arm directed at the receiver. Facial expressions were coded using a modified version of the human Facial Action Coding Scheme (FACS) [78] developed to identify facial movements of chimpanzees (chimpFACS) [79]. The *bared-teeth* face, with the mouth either closed or slightly opened and the mouth corners laterally retracted and teeth fully exposed, was identified in addition to the *funneled-lip* face, consisting of an open, rounded mouth with protruded lips. When one of the gestures was combined with either of these facial expressions, this was considered a gesture-facial expression combination. When the gesture was used without a facial expression, the face was coded as neutral. Facial expressions produced in isolation, without an accompanying gesture, were not included. The social context of the interaction was coded as either positive (e.g. greeting, grooming, play) or negative (e.g. physical conflicts, harassment). The social relationship between the sender and receiver was considered by coding whether signals were directed towards a lower- or higher-ranking individual. Finally, the outcome of the interaction (i.e. the response of the receiver) was classified as either affiliative (receiver approaches the sender and shows behaviours such as embracing, grooming or play) or avoidant (receiver is avoiding or ignoring the sender, e.g. by turning away from, hitting or pushing the sender).

As noted above, in our model, the gesture and the facial expressions contribute to the utterance (the likelihood) and the social context and the relationship contribute to the prior. We assigned parameter values to each of the components of the communicative interactions. The goal was to show that by choosing intuitive parameter values, our model can give rise to the data we observed. These values range between 0 and 1 and represent the degree to which a component is indicative of a positive (affiliative; 0–0.5) or negative (avoidant; 0.5 = 1) interpretation. We assumed the stretched-arm gesture to be weakly negative (*θ*_gs_ = 0.53) and the bent-arm gesture to be weakly positive (*θ*_gb_ = 0.47). Neutral facial expressions were set to be neutral (*θ*_fn_ = 0.5), bared-teeth expressions were set to be weekly negative (*θ*_fb_ = 0.6), and funneled-lip expressions to be strongly negative (*θ*_ff_ = 0.9). A negative context was set to be negative (*ρ*_cn_ = 0.7) and a positive to be positive (*ρ*_cp_ = 0.3). Finally, we assumed that a positive reaction was likely for a dominant sender (*ρ*_sd_ = 0.25) and a negative outcome likely for a subordinate sender (*ρ*_ss_ = 0.75).

We want to highlight that even though these parameter values are inspired by prior work and common sense, they are to some extent arbitrary and should not be taken to reflect a strong commitment to the role the individual components might play in a different context. Their main purpose is to capture the idea that different components of the communicative interaction are more or less associated with a particular response. Ideally—and hopefully in future work—these parameters would be directly estimated based on a training dataset and then used to predict a test dataset. Given the size of the dataset we had available, this approach was not possible here. The code that spells out the model architecture and the processing algorithms and that can be used to reproduce the results is available in the associated online repository: https://github.com/manuelbohn/RSApes.

Based on the model and the parameter settings, we generated predictions for all possible combinations of gestures, facial expression, dominance relationship and social context. We compared these predictions to the observations made by Oña *et al*. [50]. Our model makes predictions about the receiver’s interpretation of the utterance in context. The data, however, only recorded the receivers’ reactions—as interpreted by the human coders. We assume that the receiver’s reaction is guided by their interpretation of the utterance: when inferring a negative intention, the receiver shows an avoidant reaction and when inferring a positive intention, they show an affiliative reaction. Thus, for the purpose of the model comparison, we assume a one-to-one mapping between the interpretation of the sender’s message and the receiver’s reaction.

**Figure 2.**
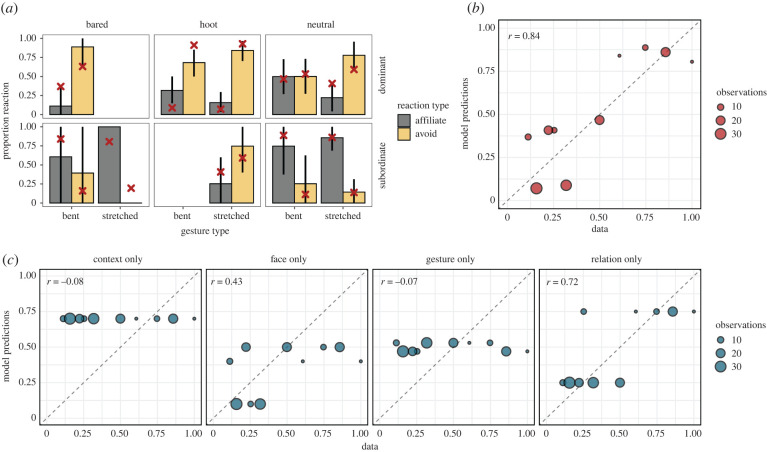
Model predictions compared to data from [50]. (*a*) The mean proportion (bars) of affiliative and avoidant reactions for combinations of gesture, facial expression, relationship and social context in the data. Only combinations with more than five observations are shown. Error bars are 95% confidence intervals based on a non-parametric bootstrap. Red crosses show model predictions. (*b*) Correlations between model prediction and data for avoidant reactions. The size of each point is proportional to the number of observations for a particular combination in the data. (*c*) Correlations for reduced models that focus only on a single component (with all other parameters set to 0.5). (Online version in colour.)

Observations in the data were not equally distributed across all possible combinations. To evaluate the model predictions, we focused on combinations that had at least five observations. All combinations that fulfilled this criterion were observed in a negative social context. When we compare the model predictions to the data, we therefore only visualize the negative context (figure 2). Note, however, that our model also generated predictions for the positive context.

In figure 2, we can see that the full model explains the data well, both quantitatively and qualitatively. The model predictions go in the same qualitative direction as the data, predicting more negative reactions when more were observed. Furthermore, many of the model predictions also align quantitatively with the data, resulting in a high correlation between the two (figure 2*b*). Let us take a closer look at some of these patterns. In most cases, the qualitative pattern in the data was the same for both gesture types. For example, in a negative context (figure 2 only includes the negative context), with a subordinate sender and a neutral facial expression, no matter if a bent or a stretched-arm gesture was used, there were more affiliative reactions. Our model predicts this pattern despite the fact that we took the stretched-arm gesture to be associated with a negative intention. The reason for this is that both gestures were assumed to have weak meanings. As a consequence, they had very little predictive power when a different, stronger information source (the dominance relationship in this case) was also available.

Next, we used this modelling framework to illustrate the theoretical point made above, namely that a focus on a single aspect of great ape communication is likely to yield an incomplete picture of the interaction. We formulated four reduced models, which use the same parameter settings as above, but selectively focused only on one of the components (all other parameters set to 0.5). When comparing the predictions from these reduced models to the data, we saw that none of them captured the data equally well compared to the full model (figure 2*c*).^3^ For example, the models focusing only on the context or the gesture completely fail to capture any structure in the data. These results, however, should be taken with a grain of salt given the—rather arbitrary—way in which we chose the parameter values. Nevertheless, we think the results nicely illustrate how computational modelling can be used as a powerful tool to study great ape communication. In the next section, we explore ways in which we can use this tool to theorize about some potential differences between ape and human communication.

## 6. Pragmatics as an amplifier

In their description of the interaction engine, Levinson & Holler [2] point out that ‘language is the tip of an iceberg riding on a deep infrastructure of communicational abilities’. Part of this deep infrastructure is pragmatics. As noted in §1, the central idea is that utterances are not interpreted at face value, but that receivers go beyond the literal and make inferences about why the sender produced a particular utterance in context. A cornerstone of this reasoning is the assumption that the sender is cooperative and informative; they produce utterances that help the receiver to infer their intention.

In the following, we enrich our model of great ape communication by pragmatics—i.e.cooperative social reasoning. From an evolutionary perspective, we may say that our great ape model stands in for the last common ancestor of great apes and humans. To recapitulate, we assume that this ancestor (and modern great apes) rationally integrated different information sources to make inferences about the sender’s intentions. This includes information contained in the utterance as well as the social context and the relationship between communicators. The pragmatic abilities are built on top of this basic infrastructure to provide modern human communication.

To evaluate this pragmatically enriched model, we want to focus on some peculiar differences that have been reported for the communicative abilities of great apes and humans. Numerous studies have shown that great apes struggle to spontaneously understand ambiguous signals, for example, pointing or novel iconic gestures [10,80–88] (with some particular exceptions [89,90]). That is, when confronted with a novel gesture or a new context, great apes usually fail to spontaneously use the gesture. These findings are peculiar because these gestures are naturally meaningful in that they either index (pointing) or resemble (iconic gestures) the referent. What is more, human children understand them spontaneously already very early in life [91–93]. Apes also seem to be somewhat sensitive to the natural meaning of these gestures. In the case of pointing, they often look in the direction the experimenter is pointing [94]. And in one study, iconic gestures were learned faster compared to arbitrary ones [95].

Why do apes struggle with spontaneous comprehension of these gestures? The results of the model above can be taken to suggest that the social context and the relationship between sender and receiver play an important role in great ape communication. In the experimental set-ups of studies on pointing or iconic gesture comprehension, these components are controlled for and therefore offer no information about the sender’s intention [10,83,86]. Great apes are left with only the gesture. If that gesture was initially only vaguely associated with one or the other outcome, it would not provide sufficient information for apes to infer the sender’s intention and thus to systematically select the referred-to object.

**Figure 3.**
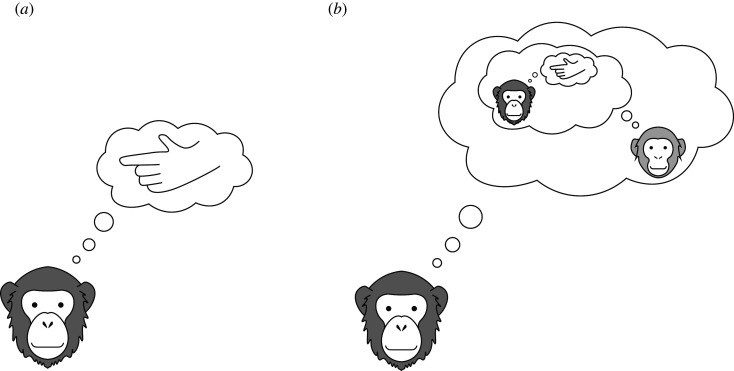
Schematic depiction of the added pragmatic reasoning component. The literal receiver (*a*) only reasons about the gesture whereas the pragmatic receiver (*b*) reasons about why the sender produced that particular gesture. The pragmatic receiver further expects the sender to produce the gesture with the goal of being informative.

Why do humans spontaneously understand these gestures? We think that the notion of pragmatics as spelled out above can act as an amplifier of vague literal meanings. That is, a human receiver assumes that the sender produced a particular gesture in a cooperative and informative manner to inform them about their intention. The additional social reasoning singles out the gesture as a communicative act that was produced with the sole purpose to express a given intention (figure 3). This line of argument is of course reminiscent of the idea that humans—but not great apes—are sensitive to cooperative communicative intentions [6]. However, we assume that pragmatic inferences are just one information source that can be exploited and that they are graded—not all or nothing. Taken together, the degree to which pragmatic reasoning amplifies a meaning depends on (a) the presence of a social reasoning mechanisms and (b) expectations about how cooperative the sender is. Next, we substantiate these ideas via our modelling framework.

The RSA framework introduced above is built around the assumptions that (a) receivers reason about why senders produce certain utterances and (b) receivers assume that senders communicate in a cooperative and informative way. This social reasoning component is formalized by embedding the model of the (zero-order) literal receiver (short-hand notation: $P_{R_{0}}$) in a model of the sender, $P_{S_{1}}$. This *pragmatic* sender chooses utterances so that they are informative for the literal receiver, while the literal receiver simply interprets utterances in line with their literal semantics. This literal receiver behaves exactly like in the great ape model (figure 3). This illustrates the way in which our model of human communication is built around our model of great ape communication. At the highest level, we now have a pragmatic receiver, $P_{R_{1}}$. These additions change our model as follows:

6.1 $$P_{R_{1}}(i|u)\propto P_{S_{1}}(u|i)P(i),$$

6.2 $$P_{S_{1}}(u|i)\propto P_{R_{0}}{\left(i|u\right)}^{\alpha }$$

6.3 $$\mathrm{and}\;P_{R_{0}}(i|u)\propto L(u,i|\theta_{u}).$$

Equation (6.2) above shows that the degree to which the sender is assumed to be informative depends on the parameter *α*. The higher *α*, the more informative the sender is assumed to be. The effect of *α*, however, depends on the presence of the sender model, which represents the additional social reasoning component that we think is characteristic of human communication.

**Figure 4.**
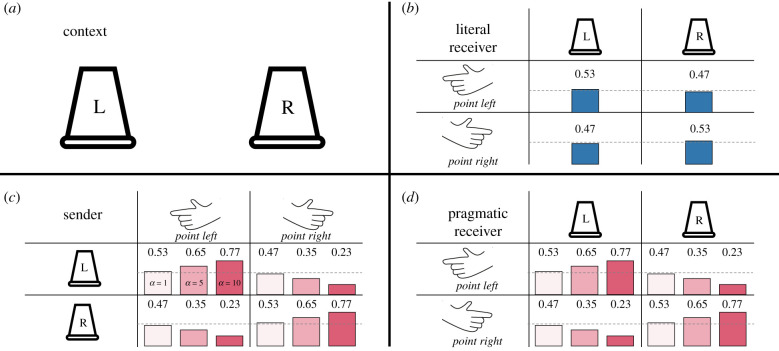
Application of the pragmatically enriched model to an object-choice task with pointing gestures. (*a*) The context with the two locations (L = left and R = right) that can be referred to. Panel (*b*) gives the interpretation probabilities of a literal receiver. (*c*) The production probabilities for the pragmatic sender for values of *α* = 1, 5 and 10. (*d*) The interpretation probabilities of the pragmatic sender based on the production probabilities in (*c*). Coloured bars visualize the probabilities in reference to chance (grey dashed line). Different shades in (*c*,*d*) correspond to the magnitude of *α*. (Online version in colour.)

When we adapt such a model to a situation in which the receiver is faced with a vaguely meaningful gesture (e.g. a point or an iconic gesture; *θ*_u_ = 0.53) without any additional contextual information, we see that the literal interpretation of the gesture simply reflects this vague meaning (figure 4*b*). We also see that pragmatic reasoning amplifies the initially vague meaning (figure 4*d*). As noted above, this is not due to the additional social reasoning component alone but critically depends on the receiver’s expectation about cooperative communication (the parameter *α*, figure 4*c*). This highlights the graded relation between assumptions about cooperativeness and pragmatic inference. Once again, we would like to point out that the specific parameter values we picked here are arbitrary and do not reflect a strong commitment to how great apes or humans interpret pointing gestures. They simply serve to illustrate the point that pragmatics may amplify vague natural meanings.

## 7. Implications and future directions

With the modelling exercise presented above we had two overarching goals. The first was to show that great ape communication is best thought of (and studied) as a multi-faceted, multi-modal, social inference process. We saw that the outcome of a communicative interaction was best predicted when signals, as well as contextual components, were taken into account. We do not say that studying these components in isolation is fruitless, but we do emphasize that focusing exclusively on, for example, the gesture or vocalization produced makes it less likely that the unfolding interaction will be understood. From our perspective, the different components play complementary roles in an integrated inference process.

Our hope is that our model proves to be a useful tool—or at least an inspiration—for future research. The approach by Oña *et al*. [50], in which many different aspects of a communicative interaction are coded, seems to be especially promising. Such work could easily be done using already existing video recordings. Models like the one presented here could then be used to specify how the different components work together. In addition, our framework provides a new way to test competing hypotheses. Instead of relying on qualitative predictions, alternative hypotheses can be formalized as alternative models and then directly compared in a quantitative way. Across studies, it would be interesting to see if general patterns emerge. For example, models that emphasize social-contextual components could make better predictions compared to models emphasizing information provided by the utterance. Or models prioritizing facial expressions could be found to outcompete models that more strongly emphasize gestures. Or *vice versa* in both cases. Experimental studies could gradually vary the information provided by signals and the social context to examine how they trade-off with one another. Such an approach might reveal quantitative differences between humans and other primates where we currently assume qualitative ones. In all of this, we think that the study of great ape communication would benefit from an interdisciplinary approach in which computational modellers work together with primatologists and comparative psychologists. Hopefully, this will allow the field to move away from asking somewhat artificial questions about the importance of individual gestures, facial expressions or vocalizations and instead move towards more comprehensive theories of the actual processes that underlie communicative interactions.

We see our model as a first step that needs to be expanded in the future. The process that we capture in our model is in-the-moment comprehension, which is only a part of communicative interaction. An easy extension would be to look at the sender: we assume our model to be symmetric and so it could be easily used to generate predictions about what types of gestures, facial expressions and vocalizations the sender should produce in different contexts given the intention they want to communicate. Furthermore, it would be interesting to extend our model to capture the temporal dynamics of communication—that is, to include mechanisms that are used to clarify or emphasize a message. Candidate behaviours in primates could be acts of persistence, repetition or elaboration that are often seen in naturalistic and experimental settings [29,35]. Including this aspect might have consequences for the cognitive architecture of the model. For example, van Arkel *et al*. [96] have suggested that a simple repair mechanism drastically changes the computational demands in human communication.

Our second goals was to demonstrate how pragmatic reasoning can act as a gradual amplifier for signals with vague meanings. This perspective might be helpful for theorizing about the gradual transition from animal to human communication. For example, Sterelny [22] has argued that the transition from animal to human communication involved shifting from code-based to ostensive inferential communication [22,97]. During this process, the tight signal–response coupling characteristic for code-based communication was loosened. This brought an increase in flexibility, allowing senders to use the same signal for different and potentially novel purposes. However, it also introduced ambiguity to the signal, which, according to Sterelny, was compensated by relying on social reasoning processes. This transition shifted the locus of selection from specific signal–response couplings to communicative behaviour more broadly, with downstream consequences for other forms of cooperative interaction [9]. Our model formalizes the trade-off between ambiguity in the signal—which is characteristic of human communication [21,98]—and social reasoning. As such, it could be used as a starting point to formalize the gradual evolution of human ostensive-inferential communication.

The gradual emergence of pragmatic social reasoning in the evolution of human communication might have had further downstream consequences for the emergence of conventional communication systems. Recently, Hawkins *et al*. [99] embedded an RSA model of pragmatic in-the-moment inferences in a model of convention formation and showed how signals with vague meanings can give rise to conventional communication systems. The meaning of a signal can get fixed (e.g. further amplified) when it is repeatedly used within dyadic communicative interactions. Conventions form when partner-specific communicative conventions are gradually transferred, via a hierarchical Bayesian model, to novel communicative partners. Work by Woensdregt *et al*. [100] suggests that the presence of conventional communication systems further facilitates in-the-moment inferences about communicative intentions, leading to a cascading coevolution of conventional communication systems and social reasoning.

Finally, our modelling approach informs discussions about the modality in which human language has evolved. For decades, there has been a strong divide between researchers arguing for a vocal or a gestural origin of language [20,47,52,101]. Recently, the idea that language origins were multi-modal has gained traction [47,101]. Our model provides a way of thinking about multi-modal communication. The model does not make any principled distinction between different modalities: for every signal, it simply asks how indicative it is for different intentions the sender might have. This explains how different signals influence each other during in-the-moment comprehension and could also be used to investigate how the burden may have shifted between modalities during the course of evolution.

## 8. Conclusion

Inspired by work on the human interaction engine, we have described a computational approach for how to study great ape communication in context. Our model assumes that great apes rationally integrate different information sources to make inferences about the intention behind a sender’s utterance in context. Using existing data, we have shown that our model makes accurate predictions about the outcome of multi-modal communicative interactions between chimpanzees in different social contexts. Based on the idea that pragmatic reasoning—social reasoning paired with assumptions about cooperative communication—acts as an amplifier for vague meanings, we suggested an explanation for some peculiar differences between the ways that great apes and humans interpret ambiguous signals. This approach illustrates some deep similarities between human and great ape communication, but also specifies in what way the human interaction engine might be equipped with some special parts.

## Data Availability

All data and model code are available in a public online repository: https://github.com/manuelbohn/RSApes.

## Endnotes

1 The RSA framework usually uses *speaker* and *listener* to describe the agents involved. Here, we continue to use the terms *sender* and *receiver* instead to be more inclusive of non-human and human multi-modal communication.

2 We are grateful to David Leavens for pointing us to Altmann’s work.

3 In the online repository, we also include a model in which the strength of the meaning of gestures and facial expressions was switched. That is, gestures were assumed to have a rather strong meaning and facial expressions a weak one. This model makes worse qualitative and quantitative predictions compared to one presented in the paper.
